# Coronary Vasculature in Cardiac Development and Regeneration

**DOI:** 10.3390/jcdd5040059

**Published:** 2018-12-17

**Authors:** Subir Kapuria, Tyler Yoshida, Ching-Ling Lien

**Affiliations:** 1 The Saban Research Institute of Children’s Hospital Los Angeles, Los Angeles, CA 90027, USA; tkyoshida@usc.edu; 2 Department of Biological Sciences, Dornsife College of Letters, Arts and Sciences, University of Southern California, Los Angeles, CA 90007, USA; 3 Department of Surgery, University of Southern California, Los Angeles, CA 90033, USA; 4 Department of Biochemistry & Molecular Biology, Keck School of Medicine, University of Southern California, Los Angeles, CA 90033, USA

**Keywords:** heart regeneration, coronary vessels, endothelial cells, perivascular cells

## Abstract

Functional coronary circulation is essential for a healthy heart in warm-blooded vertebrates, and coronary diseases can have a fatal consequence. Despite the growing interest, the knowledge about the coronary vessel development and the roles of new coronary vessel formation during heart regeneration is still limited. It is demonstrated that early revascularization is required for efficient heart regeneration. In this comprehensive review, we first describe the coronary vessel formation from an evolutionary perspective. We further discuss the cell origins of coronary endothelial cells and perivascular cells and summarize the critical signaling pathways regulating coronary vessel development. Lastly, we focus on the current knowledge about the molecular mechanisms regulating heart regeneration in zebrafish, a genetically tractable vertebrate model with a regenerative adult heart and well-developed coronary system.

## 1. Introduction

The heart pumps blood relentlessly during an animal’s lifespan and is critical for survival. Such functional load demands a continuous supply of nutrients and oxygen in heart muscle cells, cardiomyocytes. The coronary circulatory system made of coronary arteries, veins and capillaries meets this demand and provides a route for immune cells and other regulatory cells to constantly maintain heart function while protecting the heart from physiological and mechanical challenges. Thus, it is not surprising that coronary artery disease is the leading cause of human mortality [1]. In adult mammals, inhibition of blood flow to the cardiomyocytes immediately cause cell death and impose irreversible damage to the heart wall. Surprisingly mammals at the neonatal age [2,3] and many adult non-mammalian vertebrates (e.g., zebrafish, newts) [4,5,6,7,8] have remarkable ability to repair and regenerate damaged hearts and thus drew significant attention over the past decades for exploration of the underlying mechanisms of this process. Heart regeneration requires coronary vessel formation in the regenerating area. Two recent studies on zebrafish [9] and neonatal mice [10] demonstrated that vascularization begins at a very early stage of heart regeneration and is critical for cardiomyocyte proliferation and healing. Heart regeneration can recapitulate the processes that occur during heart development. In this review, we will summarize the cell types and signaling pathways involved in coronary vessel development with a focus on non-mammalian models. We will compare these processes with revascularization during heart regeneration.

## 2. Evolutionary Aspects of Coronary Vessels

The coronary circulatory system is dispensable in many non-mammalian vertebrates [11]. In all vertebrates, the heart begins to develop as an avascular heart tube. Heart development is a classic example of ontogeny following phylogeny. The coronary circulatory system evolved gradually with the increased complexity of the cardiomyocyte layers. The early vertebrate heart is mostly made of the spongy trabecular cardiomyocytes. In the trabecular structure, the sponge-like sinusoidal organization of the cardiomyocytes has increased surface area which allows efficient distribution of nutrients and oxygen to individual cardiomyocytes through diffusion from luminal venous blood. These organisms have low metabolic demands, and a low nutrient and oxygen circulation rate can meet this need. Thus, although the hearts with trabecular cardiomyocytes have lower cardiac output, they can still serve the purpose [11]. As the vertebrates evolved from the agnathans (jawless) to the gnathostomes (jawed vertebrates) with increased metabolic demand, the need for increased cardiac work rate made the heart gradually stronger with increased thickness of the densely packed cardiomyocyte layers, the compact myocardium. In these hearts, simple diffusion is not sufficient for the nutrient and oxygen distribution, and they developed a dedicated coronary circulatory system to serve cardiomyocytes in the compact layers [11].

**Figure 1:**
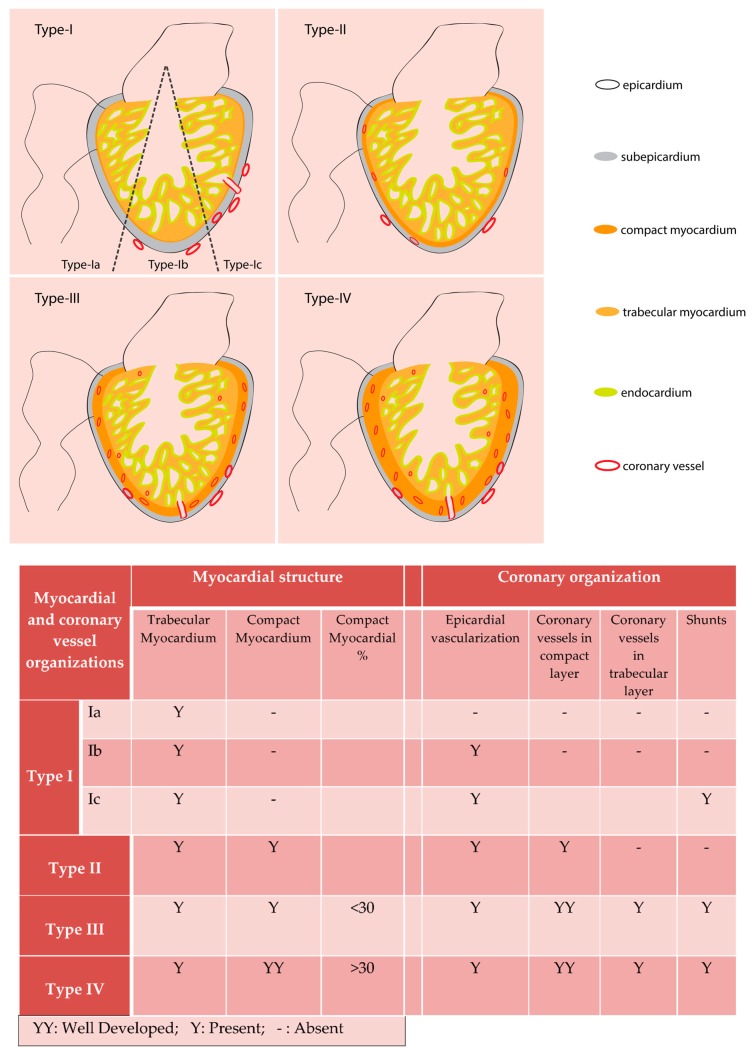
Fish ventricle classification following Tota (1989). Based on cardiomyocyte composition and coronary vessel organization fish hearts are classified into four types. Type I ventricle is mostly made of trabecular myocardium and have superficial epicardial coronary vessels (Type Ib and Type Ic). Type II hearts have ventricles with coronary vessels only in the thin, compact myocardial layer. Type III and Type IV hearts have well developed compact myocardium and have coronary vessel distribution in the compact and trabecular myocardium. Type III and Type IV hearts consist of <30% and >30% compact myocardium respectively.

Not all fish species have blood flow from coronary circulation to the trabecular cardiomyocytes [12]. Tota in his work with fish, classified hearts into four different types, based on the level of the ventricular cardiomyocyte compaction and vascularization. The Type-I heart ventricle has only trabecular cardiomyocytes and consists of three subclasses. The subclass Type-Ia hearts are avascular (e.g., *Scorpaena*), and the Type-Ib hearts have non-penetrating superficial vessels on epicardium (e.g., *Pleuronectes*). The subclass Type-Ic hearts have the epicardial vessels, which enter subepicardial space and emptied in intratrabecular luminal space (e.g., hemoglobin-less Antarctic icefish) [12]. In all the Type-I hearts, the systemic luminal blood, with low oxygen partial pressure supply oxygen to the cardiomyocytes. Many fish species have a Type-I heart. According to several studies, ~80% of the studied fish species [13,14] or around 50% of teleost species [11] have the Type-I ventricle. The other three categories of the hearts have a dedicated coronary circulatory system, which supplies oxygen to cardiomyocytes from blood with comparatively higher oxygen partial pressure than the systemic blood. The Type-II heart ventricle is mostly made of spongy trabecular cardiomyocytes and has a thin layer of compact myocardium. In these hearts, coronary circulatory vessels are present in the compact muscle layer but not in the trabeculated myocardium (e.g., *Conger*). Both of Type-III and Type-IV have circulatory vessels in both compact and trabeculated myocardial layers, but they differ in the amount of cardiomyocyte compaction. The compact cardiomyocyte form <30% of the total heart mass in Type-III heart. Most elasmobranchs have a Type-III heart. The Type-IV heart has compact myocardium >30% of the total heart mass (summarized in Figure 1). Fish such as tunas and endothermic sharks are under the Type-IV category [11]. From such classification, it appears the vertebrate heart evolution took different routes in elasmobranchs and bony fishes. While elasmobranch hearts develop vascularization in the trabecular layer, some bony fishes probably do not maintain this vasculature in the trabecular layer while others do [15]. Further studies are needed to understand the relationship between heart size, structure, myocardial composition, and the patterns of coronary vessel formation in teleosts.

It is hypothesized that in the early vertebrates, coronary vasculature evolved from pronephric external glomeruli (PEG). Highly vasculogenic glomerular cells transferred to the heart and supplied it with blood vessel progenitors. Hypothetically, this could be the first step in evolution for myocardial vascularization. PEG is found in the representatives of the most primitive vertebrates (e.g., Lamprey). During evolution, the anterior-most part of pronephros disappeared, and the cardiac inflow tract (sinus venosus) and liver expanded. With time, a temporary embryonic structure, proepicardium (PE) emerged. Such evolutionary origin may be reflected in the developmental association between PE/PEG and liver/sinus venosus in the gnathostomes [16]. PE is an extracardiac cluster of cells developmentally originates as a coelomic outgrowth and located dorsally to the heart tube between developing liver and sinus venosus [17,18,19]. During development, PE cells migrate to the heart and form the outer epithelial layer, epicardium. Epicardium plays an essential role in coronary vasculature development [17,18,19]. Several genes commonly expressed in the kidney and the PE including the transcription factors Wilms tumor 1 (*Wt1*), epicardin/capsulin (*Tcf21*), T-box factor 18 (*Tbx18*) supporting such developmental and evolutionary link between PE and kidney [18].

From the early tubular structure, the heart gradually increases the myocardial layers to match the increased blood circulation demand in a rapidly growing embryo. At this stage, the myocardial growth occurs by cardiomyocyte proliferation (hyperplasia). In endothermic vertebrates (e.g., birds and mammals), the heart initially becomes a Type-I like heart, with avascular trabecular myocardium and very thin mantle of compact cardiomyocytes. The emergence of the coronary circulatory system is a well-regulated combination of several processes, which include angiogenesis vasculogenesis, arterial development and maturation and correlates with the timing of myocardial growth [20]. The developing coronary plexus eventually forms organized capillary networks covering and penetrating the myocardial layer. The coronary artery eventually grows towards the aorta and penetrates at the root of the aorta. At this point, blood flow begins through the coronary circulatory system [21].

From the regenerative perspective, the vertebrate heart categorization based on myocardial and coronary vasculature [12] is alluring to find a correlation between the structural simplicity and regenerative ability. However, the findings in the bony fish medaka and zebrafish made such correlation more complicated. While the zebrafish heart can regenerate remarkably even after removing ~20% of the ventricular mass, the medaka lacks such ability and they are more susceptible to the lethality caused by heart amputation [22,23]. Interestingly, medaka do not have coronary vessels and there are no vascular cells observed in the wound area post amputation [22,23]. Such phenomena warrant a detailed understanding of the cellular and molecular mechanisms of the roles of coronary vessels and heart regeneration. The tissue regeneration follows/reactivates similar mechanisms those occur during the tissue development. Studying the development of the coronary circulatory system along with the heart development may shed some light in our understanding of the mechanisms of how coronary circulatory system participate in heart regeneration.

## 3. Endothelial Cells: Origins and Developmental Signaling Mechanisms

The principal cellular components of a blood vessel include the endothelial cells and the mural/perivascular cells. Endothelial cells form the vessel wall, and the mural cells encircle the endothelial wall on its abluminal surface and regulate blood vessel development/maturation, stability, and the contractile activity [24].

The origin of the coronary endothelial cells has been the subject of interest for a long time. The findings in this aspect evolved and contradicted previous findings as the employed technologies improved over time, and different model organisms showed different origins. Based on the morphological studies, initially, the coronary arteries and veins were believed to sprout from the aorta and sinus venosus respectively [25,26] by endothelial budding. Later extensive studies were performed in the avian model organisms (e.g., chick, quail). Several lineage-tracing approaches were used, such as ink-injection in the specific area of the embryo at a particular developmental stage and following the lineage. In some studies, the lineages were analyzed by retroviral (replication-defective virus expressing the marker gene, e.g., β-galactosidase) tagging or the chick-quail chimera formation by transplanting specific area of the chick embryo with the equivalent quail tissue and follow the lineage with quail specific antibodies. Some findings in these studies negated the possibility of endothelial budding of the coronary arteries from the aorta, instead described penetration of the developing arteries to the aorta [27,28]. Vasculogenesis was suggested as the mechanism of coronary circulation development in chick embryos [28]. In a chick-quail chimera analysis, the extracardiac origin of the coronary endothelial cells was described, and pure epicardial primordium transplant was shown unable to contribute in coronary endothelial cell development [29]. Later, another study with improved orthotopic transplantation techniques showed the quail proepicardial villi transplant in the chick embryo contributed significantly in coronary endothelium development [30]. Several other studies using alternative approaches such as retroviral or fluorescent labeling of the epicardial mesothelial cells or the matrigel culture of the proepicardial cells reported the contribution of epicardial-derived cells (EPDCs) in coronary endothelial cell formation [31,32,33].

In mice, advanced genetic lineage-tracing studies by utilizing the Cre-LoxP based method demonstrated that the sinus venosus and endocardium mostly contribute to forming coronary vasculature in a complementary fashion in different regions of the heart [34,35,36,37,38]. The *Sema3D+* and *Scx+* PE cells have lesser contribution to coronary endothelial cells than the subepicardial endothelial precursor from sinus venosus and the endocardium. Furthermore, *Sema3D+* and *Scx+* PE cells can contribute to early sinus venosus and endocardium [35]. There are not many studies about coronary endothelial development in reptiles and amphibians. Urodele amphibian such as newts and anuran amphibians lack coronary vasculatures [39,40,41,42] as a possible consequence of a well-developed buccopharyngeal and cutaneous respiration system. Some amphibians develop a vestigial surface coronary vessel on the outflow tract [43].

In fish, there are a limited number of studies on coronary endothelial cell development. In sharks, coronary vasculature develops at the embryonic stage. In a dogfish study, the coronary veins first appeared as a diverticulum of the sinus venosus [44]. Later, it was suggested that the sub-epicardial mesenchymal cells form capillary-like structures, which finally coalesce and form the cardiac vein precursors indicating in situ vasculogenic origins of the cardiac veins [45]. Unlike sharks, birds or mammals, in zebrafish coronary vasculature originate during post-embryonic development. The coronary vasculature formation initiated 1–2 months after hatching when juvenile ventricular myocardium expansion has started to form cortical cardiomyocytes [46,47]. The coronary endothelial cells emerge as the angiogenic sprouting from the endocardial derived cells at the atrioventricular (AV) canal. Lineage tracing approach and multispectral clonal analysis demonstrated that multiple AV endocardial cells migrate to the heart surface and form the coronary vasculature. Unlike mammals, zebrafish lack contribution from sinus venosus in coronary vessel formation. Sinus venosus and atrium remained avascular throughout the development and in adults [47]. Therefore, AV canal endocardium is the primary source of coronary endothelial cells in zebrafish heart. The origin of the coronary endothelial cells of the giant danio, a close relative of zebrafish, is somewhat different. The coronary vasculature in giant danio first appears in the late larval stage. By BS lectin staining, coronary vessels were shown to emerge from both bulbus arteriosus (BA) and AV canal. The first emergence of the vessel preferentially occurs as an extension of the hypobranchial artery (from bulbus arteriosus). In this study another fish of distant clade, blue gourami, showed the first appearance of coronary endothelial cells as an extension of one or many hypobranchials (a vessel plexus from proximal bulbus) [48]. Together, these findings indicate that fish can follow diverse paths for developing coronary vasculature and these processes are likely influenced by the structure of the heart and the need of the myocardium. The origins of the coronary endothelial cells in different organisms are summarized in Table 1.

### Signaling Pathways Regulating Coronary Endothelial Cell Development

Origin and development of the coronary endothelial cells are regulated by the interaction between the cardiomyocyte layer, myocardium, and the flanking epithelial layers, epicardium, and endocardium. During development as the expanding myocardial layer become hypoxic it activates Hypoxia-inducible factor, HIF-1α in avian and mammalian embryos [50]. In the developing chicken heart during ventricular septation, the hypoxia indicator EF5 stained outflow tract (OFT), AV junction, a portion of interventricular septum (IVS), a discrete region of the atrial wall and the ventricular myocardial wall periphery. HIF-1α nuclear localization co-localized with EF5 staining indicating HIF-1α activation in some of these regions [51]. Among several genes activated by HIF-1α, Vascular endothelial growth factor (VEGF) is an essential regulator for endothelial cell differentiation, vessel development by angiogenesis and vasculogenesis [52,53,54,55,56]. In mouse embryos, around the similar developmental stage, strong *Vegfa* expression was found in the ventricular and OFT myocardium, IVS, and at the AV junction [57,58] which is strikingly similar to EF5 and HIF-1α activation pattern [51] indicating a possible link between the hypoxic condition and VEGF activation. The interaction between the myocardially expressed VEGFA, and corresponding endocardial VEGFR-2 expression was demonstrated to contribute in coronary plexus formation that matures into intramyocardial coronary arteries in the mouse embryo [36]. In another study, epicardially expressed VEGFC was suggested to interact with the receptors VEGFR-2 and VEGFR-3 on coronary endothelial cells and VEGFR-2 on sinus venosus [38]. This interaction drives sinus venosus derived subepicardial coronary vessel development. Thus in mice, VEGFA and VEGFC regulate the coronary vasculature development in a complementary fashion by promoting intramyocardial and subepicardial coronary vasculatures respectively [38]. In zebrafish, *vegfaa* normally express in the epicardium [59]. The *vegfaa^−/−^* mutant embryos show severe angiogenic defects. In the *vegfaa^−/−^* mutant fish rescued (by injecting *vegfaa* 121 or *vegfaa* 165 mRNA at the one-cell stage) to adulthood, the coronary vessels are irregularly distributed and thinner compared to wild-type fish [9].

Fibroblast growth factor (FGF) is another signaling pathway that acts along VEGF in regulating coronary vasculature development. In a chick-quail chimera study when the transplant was preincubated with FGF and VEGF, the local vascularization of the compact myocardium increased significantly [60]. Retinoic acid induces FGF ligands expression in the epicardium [61,62]. Epicardial FGF then induces epicardial epithelial to mesenchymal transition (EMT) and Wnt ligand expression. In the chick embryo, the epicardial overexpression of WNT9b or activated β catenin caused increased vasculogenesis [62]. In the mouse embryo, epicardial and endocardial FGF ligands also indirectly regulate coronary vessel growth by signaling to cardiomyocytes through the receptors FGFR1 and FGFR2. Myocardial FGF signaling induces a wave-like activation of the Hedgehog (HH) signaling in the myocardium from the AV groove to the ventricular apex between embryonic stage E12.5 to E13.5. Among the HH ligands (SHH, IHH, DHH), Sonic hedgehog (SHH) expresses in the epicardium and upregulates the receptor Patched-1 (*Ptc1*) expression in the myocardium. HH signaling in the myocardium is required for myocardial expression of *Vegfa*, *Vegfb*, and angiopoietin-2 (*Ang-2*) and likely the expression of *Vegfc* in perivascular cells and these factors regulate coronary vascularization [63]. Myocardium derived angiopoietin-1 (*Ang-1*) promotes coronary vein formation. The subepicardial venous structures originate from the sinus venosus in mouse embryos. ANG-1 regulates the migration of APJ (also known as Apelin receptor)-negative immature endothelial cells from sinus venosus into the atrium and ventricular myocardium. These immature endothelial cells from the sinus venosus express TIE-2 receptor. When these endothelial cells enter myocardium, TIE-2 is activated by ANG-1 and promotes the endothelial cell migration, proliferation and differentiation into coronary veins [64].

In juvenile zebrafish, the Cxcr4-Cxcl12 chemokine signaling regulates coronary angiogenesis. During zebrafish heart development, cardiomyocytes express the ligand Cxcl12b and guide Cxcr4a expressing endothelial cells to undergo angiogenesis to cover the ventricular myocardium [47]. Consistent with the finding in zebrafish, CXCR4 has been identified as a late arterial gene by single cell RNAseq in mouse embryos, and the CXCL12/CXCR4 signaling axis has been shown to regulate DACH1 stimulated shear-guided endothelial cell migration and coronary artery growth [65].

Coronary vessel formation is a versatile process that can adapt to the need of the myocardium. Different signaling pathways have been discovered to regulate coronary endothelial cells originating from different sources in mice. ELABELA (ELA)-APJ signaling axis is mainly required for the sinus venosus derived coronary endothelial cells in mouse embryos. When this developmental process is compromised, endocardium derived endothelial cells expand to compensate the lost sinus venosus derived cells to ensure normal heart development [66].

## 4. Mural Cells: Origin, Development and Signaling Pathways in Recruitment and Differentiation

Mural cells/Perivascular cells (vascular smooth muscle cells and pericytes) in the coronary vasculatures have a unique developmental origin. Epicardium, the outermost epithelial layer of the heart, plays essential roles in the coronary vessel development. In early development, epicardium is formed from the PE cells that migrate to the surface of the heart. PE is highly conserved during the vertebrate heart development [67,68,69,70,71,72,73,74]. PE cells form a bilateral cluster in zebrafish at around 48 h after fertilization [67]. During chick and *Xenopus* development, PE emerges asymmetrically and develops only from the right side around Hamburger-Hamilton stages 14, and stage 41 respectively [68,75]. The migration of the PE cells towards the developing heart is distinct in zebrafish and chick/frog (*Xenopus*) embryo. In zebrafish, pericardial flow generated by heartbeat promotes the release of PE cells from the lining of the pericardial cavity and directs the motion of the PE cells and their colonization of the myocardium [76]. In chick and frog, PE cells migrate to the heart surface by a unilateral tissue bridge formation [68,77]. After reaching the heart, the PE cells proliferate and cover the myocardium forming the epicardial epithelium [76,77].

In an effort of fate mapping the proepicardial descendent cells, in the chicken embryo, the vital dye Dil or replication defective retrovirus (β-galactosidase encoding) tagged PE cells were found to form coronary smooth muscles, perivascular connective tissue and endothelial cells. The study led to the conclusion that the coronary smooth muscle fate was predetermined in PE before their migration to the developing heart [31]. Later, by using in vitro culture of the quail epicardial cells and the chick-quail chimera study, it was shown that the epicardial cells undergo EMT generating subepicardial mesenchymal cells, which further differentiate into coronary smooth muscle, perivascular fibroblast and intramyocardial fibroblast [78]. The subepicardial mesenchymal cells generated from the epicardium by EMT are EPDCs [79,80]. In vivo, EPDCs migrate into the subepicardial space and then to myocardium before further differentiation [81].

The growth factor FGF, EGF and VEGF treatment to the cultured quail epicardial cells induces them to take the mesenchymal fate [78]. Based on these findings, it was hypothesized that in the AV canal, where the myocardium is flanked both subepicardially and subendocardially by the mesenchymal matrix, the myocardial-secreted growth factors could induce both epicardial to mesenchymal as well as endocardial to mesenchymal transformation. It was further postulated that from nascent vascular endothelial cells, the secreted factors like EGF and PDGFBB could associate subepicardial mesenchymal cells with the endothelial cells to form mature vessels [78]. Recently in the mouse embryo, coronary smooth muscle cells and pericytes were also shown to originate from endocardial endothelial cells by endothelial to mesenchymal transition. At midgestation stage, mural cell precursors were mostly observed in AV canal and OFT [82]. In zebrafish, elegant genetic lineage tracing demonstrated that *tcf21* positive epicardial cells contribute to the perivascular cells [83]. In another study, using a cryoinjury model, EPDCs were shown to differentiate into myofibroblasts and perivascular cells [84].

### Signaling Pathways Regulating Coronary Perivascular Cell Development

Several cell-signaling mechanisms have been identified regulating mural cell recruitment on endothelial cells and their differentiation. Platelet-derived growth factor signaling is a major pathway, which regulates mural cell recruitments. Endothelial cells of the angiogenic vessel secrete the ligand PDGFB that binds with the receptor PDGFRβ on the pericyte surface [85,86]. PDGF signaling also regulates epicardial to mesenchymal transition of EPDCs, their migration, and development of coronary smooth muscle cells [87,88]. Interestingly, it was recently reported that pericytes encircling small coronary vessels are the progenitors of the coronary artery smooth muscles [89]. Therefore, the roles of PDGFRβ signaling in mouse coronary smooth muscle cell formation is likely due to its functions in pericytes.

Notch signaling regulates mural cell differentiation depending on mural cell-endothelial cell contact. In the epicardial Notch loss of function mouse (*Wt1-Cre: Notch^flox/flox^*) coronary artery development and perivascular cell differentiation were impaired [90]. In another study, epicardial deletion of *Rbpj*, the notch signaling transcriptional regulator, abrogated smooth muscle cell differentiation from EPDCs and conditional Notch over-activation caused premature differentiation of smooth muscle cell [91]. It was also demonstrated that Notch signaling acts upstream of the TGFβ and PDGF signaling in regulating coronary smooth muscle cell differentiation [91]. Blood flow plays a significant role in smooth muscle cell differentiation. The shear stress generated by blood flow induces the Notch ligand Jagged-1 expression in the endothelial cells that activates Notch 3 receptor expressed on the surrounding pericytes at the arterial remodeling zones. Notch activation in pericytes promotes their differentiation into smooth muscle cells. Thus, blood flow regulates coronary vessel maturation by Notch-mediated smooth muscle cell differentiation [89].

## 5. Coronary Vasculature and Cardiac Regeneration: Signaling Mechanisms

**Figure 2:**
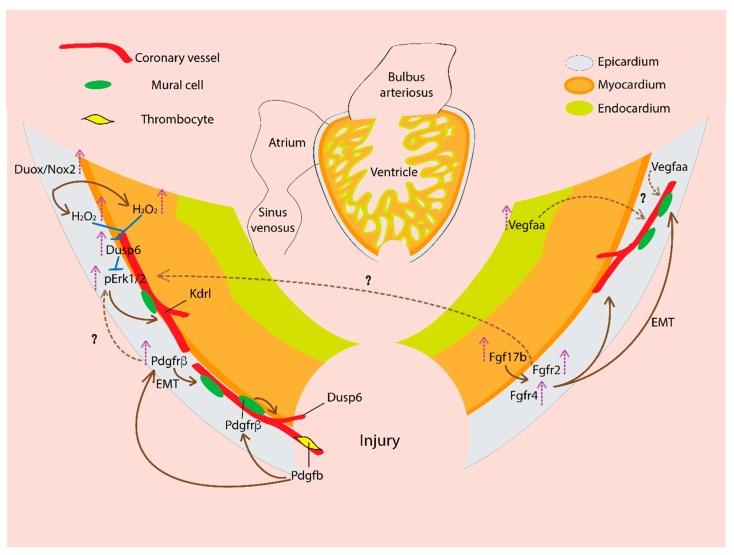
Signaling pathways regulating new coronary vasculature formation during zebrafish heart regeneration. In a regenerating heart, vascular endothelial growth factor (VEGF) ligand, Vegfaa expression increases and is required for coronary vascularization. Vegfaa expresses in the epicardium, and upon injury, Vegfaa expression is upregulated in the endocardium. Fibroblast growth factor (FGF) ligand, Fgf17b expression increased in the myocardium, and the receptor Fgfr2 and Fgfr4 are expressed in the epicardium. FGF activity drives epithelial to mesenchymal transition (EMT) of the epicardial cells, which generate mural cells. In the regenerating heart, FGF signaling promotes neovascularization. Platelet-derived growth factor (PDGF) ligand, Pdgfb is likely expressed by thrombocytes (platelets). Pdgfb activates Pdgfrβ the receptor on mural/perivascular cells and drives their recruitment to the growing vessels and thus supports coronary vessel formation. The Pdgfrβ expression is upregulated in the injured heart epicardium. PDGF signaling promotes EMT of the epicardial cells, which generate mural cells and contribute to coronary neovascularization. Duox/Nox2 expression increases in the epicardium which generates H_2_O_2_. H_2_O_2_ inhibits Dusp6, an inhibitory phosphatase of pErk1/2 MAP kinase and thus increase pErk1/2 activity, which promotes *kdrl*+ coronary vessel formation. The Dusp6 expression is induced in injured heart. Dusp6 expresses in the epicardium, coronary endothelial cells, and myocardium. The nascent coronary vessels in the regenerating area express Dusp6. Dusp6 inhibits pErk, which could act downstream of growth factors like PDGF, FGF. Thus, Dusp6 attenuates pErk mediated growth factor responses in a regenerating heart.

Coronary vascularization is an essential part of heart regeneration. In zebrafish, within 15 h after the cryoinjury of the adult heart, blood vessels begin to grow inside the wound area. Such ability of the damaged area revascularization is temporally restricted. Revascularization of the injured area is required to support cardiomyocyte proliferation. Inhibiting the early revascularization decreases tissue regeneration efficiency, which leads to fibrotic scar formation [9]. Similar phenomena were found in mammalian heart regeneration model. In neonatal mice, within two days after apical resection, coronary capillaries began migration in the injured area and became mature artery by five days. The vascularization always precedes cardiomyocyte migration, which co-aligns with the ingrowing coronary vessels [10]. Several cellular signaling mechanisms were found to contribute in this revascularization process. Due to the scope limitation, we will only discuss the signaling pathways of the zebrafish coronary revascularization (summarized in Figure 2).

### 5.1. Vascular Endothelial Growth Factor (VEGF)

VEGF signaling regulates the fast revascularization of the wound area. In the cryoinjured heart, *vegfaa* expression increased significantly as early as one day after the injury [59]. The expression pattern is dynamic during regeneration. In *TgBAC*(*vegfaa:EGFP*) fish, it was shown that in uninjured heart *vegfaa* normally express in the epicardium. After ventricular resection, *vegfaa* expression increased in the endocardium around the wound area. The global genetic cardiomyocyte ablation induces endocardial *vegfaa* expression throughout the heart [59] indicating the expression in endocardium adjacent to the regenerating muscle.

The cryoinjured *vegfaa^−/−^* mutant heart showed delayed revascularization with a disorganized plexus formation. The ubiquitous overexpression of the dominant negative, dn-Vegfaa isoform under heat-shock 70-like (*hsp70l*) promoter severely affected revascularization of the cryoinjured area. In *Tg*(*hsp70l:dn-vegfaa*) fish, the vascular density at the injured area reduced ~75%, and this caused almost 60% decrease in cardiomyocyte proliferation at 7 days after the cryoinjury. Inhibiting early revascularization in the *dn-vegfaa* fish, changed the scar matrix composition (more collagen deposition instead of fibrin-collagen mixture) in the damaged area which eventually caused permanent collagen scar formation and incomplete regeneration [9].

### 5.2. Fibroblast Growth Factor (FGF)

FGF signaling is activated in epicardium during zebrafish heart regeneration. FGF activity promotes EMT of the EPDCs which drive coronary vascularization and resulting cardiac regeneration. In the ventricular amputation injury model, cardiomyocytes along with other cells in the injured area upregulate *fgf17b* ligand expression around day seven post-injury. This expression pattern is maintained until day 30, many cells in the regenerated myocardial wall express *fgf17b.* Epicardium around the injured area upregulates FGF receptors *fgfr2* and *fgfr4* expression following a similar timeline. Inhibiting the signaling by ubiquitous expression of the dominant-negative form of the Fgfr1 driven by the heat shock responsive *hsp70* promoter, impairs cardiac regeneration and leaves a scar at day 30 after the injury. In the *hsp70:dn-fgfr1* fish, epicardial cells fail to integrate into the regenerating tissue and the coronary neovascularization is severely impaired [92].

### 5.3. Platelet-Derived Growth Factor (PDGF)

PDGF signaling is activated in the injured area of the amputated heart. After amputation, circulating thrombocyte in the wound area expresses the ligand *pdgfb*. The receptor *pdgfrβ* expression was observed in the epicardium, subepicardium and the fibrin clot at the injury site at day six after amputation. The *pdgfrβ* expression is maintained at day 10 and day 14. In an in vitro heart explant culture system, recombinant Pdgfbb treatment induced the epicardium-derived epithelial cells to lose the cell-cell contact and undergo EMT like transition. Pdgfrβ inhibitor blocks epicardial cell proliferation, EMT, and new coronary vessel formation in the wound area. Together these findings suggest that after the cardiac injury, Pdgfrβ signaling is activated in the epicardium. The activated epicardial cells then proliferate and undergo EMT to generate mural cells and promote coronary vasculature formation [93,94].

### 5.4. Reactive Oxygen Species (ROS)

NADPH-oxidase (Nox) and dual oxidase (Duox) enzymes in the plasma membrane catalyze electron transfer from NADPH to the molecular oxygen to generate superoxide anions. Superoxide dismutase readily converts superoxide anions into hydrogen peroxide (H_2_O_2_). H_2_O_2_ is required for efficient heart regeneration after ventricular resection [95].

At day three after the injury, *duox* and *nox2* are expressed at the injury site in the epicardium. The *duox*/*nox2* expression sustains at day 7 and day 14 before it goes back to the basal level at day 30 after the injury. The *duox*/*nox2* expression generates H_2_O_2_ in the epicardium and adjacent myocardium in the injured area visualized by a fluorescent protein-based H_2_O_2_ sensor, Hyper. H_2_O_2_ oxidizes the dual-specificity MAPK phosphatase 6 (Dusp6) and promotes their degradation by ubiquitin/proteasome [95]. Dusp6 dephosphorylate pErk1/2 and inactivate them. Thus, by inhibiting Dusp6, H_2_O_2_ derepresses pro-regenerative pErk1/2 MAP kinase signal which acts downstream of the FGF and PDGF signaling in the epicardium [95,96].

By treating *Tg*(*kdrl:EGFP*) zebrafish with diphenyleneiodonium (DPI), a Duox/Nox enzyme inhibitor and BCI, an inhibitor of Dusp6 phosphatase activity, it was shown that H_2_O_2_ and Dusp6 regulate coronary vessel growth in the injured area. Inhibiting H_2_O_2_ generation by DPI treatment reduced *kdrl^+^* coronary vessel formation during heart regeneration. However, BCI co-treatment with DPI maintained *kdrl^+^* vascularization [95]. Recently, another study reported similar findings of Dusp6 in cardiac regeneration [96]. Treating ventricle-amputated zebrafish with BCI and BCI215 (BCI analog), improved regeneration by increasing cardiomyocyte proliferation and angiogenesis and by reducing fibrosis. Dusp6 expression upregulated at day one after the injury and the elevated expression sustained at day seven post-injury. Using a reporter transgenic fish driven by the *dusp6* promoter *Tg*(*dusp6:memGFP*), *dusp6* expression was observed in endothelial cells along with mild expression in the cardiomyocytes. During regeneration, nascent coronary vessels showed *dusp6* expression in the regenerates. More importantly, *dusp6* mutant hearts showed similar accelerated regeneration as the chemical inhibitor treated fish, where many fibrosis genes were downregulated. The uninjured *dusp6* mutant heart showed thicker compact myocardium with more vessels showing pErk expression. In the *dusp6* mutant fish, heart regeneration is only mildly affected Pdgfrβ signaling inhibition [96]. It remains to be determined how neovascularization in *dusp6* mutant fish is impacted by Pdgfrβ and Fgf signaling pathways.

Despite the characterization of the signaling pathways involved in new coronary vessel formation in zebrafish heart regeneration, it remains to be further investigated whether the coronary arteries regenerate the same architecture as in the uninjured hearts. Furthermore, the epicardium seems to play a significant role by interacting with other cell types (e.g., myocardial cells) in coronary vascularization of the damaged area [92,94,95]. Although during the early vascularization (~15 h after injury) EPDCs does not enter the injured area [9], the organ-wide cell proliferation and embryonic gene expression is observed in the epicardium soon after the injury [92,97,98]. Thus, the signaling pathways, which regulate epicardium during cardiac regeneration, may indirectly contribute to coronary neovascularization. The hedgehog (Hh) signaling from the cardiac outflow tract (bulbous arteriosus) regulates epicardial regeneration [99]. Myocardial inhibition of the NF-κB has a pleiotrophic effect including significantly decreasing the epicardial cell infiltration into the wound area during cardiac regeneration [100]. Insulin-like growth factor (Igf) regulates cardiomyocyte proliferation during regeneration. *igf2b* is upregulated in the endocardium and epicardium in the wound area after ventricular resection [101]. Interestingly in the cryoinjured heart, activated insulin-like growth factor receptor 1 (phosphor-Igf1r) was detected in the endothelial cells but not in the cardiomyocytes [102]. It will be interesting to explore if Hh NF-κB and Igf contribute in coronary neovascularization during repair. Retinoic acid (RA) signaling regulates coronary vasculature development in the mouse. Alteration in RA-signaling affects ventricular coverage of coronary vessels reducing their density. RA signaling is involved in vessel morphology and epicardial derived mural cell recruitment on the vessel in mouse embryos [103]. Interestingly in adult zebrafish, within 3 h after the ventricular amputation, RA synthesizing enzyme *aldh1a2* expression is strongly upregulated in the endocardium and sequentially in the atrial and the ventricular epicardium [92,104]. It remains to be determined if RA signaling plays a role in regulating coronary vessels in zebrafish. 

## 6. Conclusions and Future Perspectives

Myocardial regeneration requires the formation of the new vasculature that is essential for the survival of the cardiac tissue and to adapt to the changing environment after traumatic damage. The research utilizing different model organisms have shed light on how the natural regeneration of the heart can be achieved. Many developmental processes of the hearts are recapitulated during cardiac regeneration. The studies in non-mammalian animal models have revealed molecular mechanisms of coronary vessel development and perhaps also in revascularization. Several important questions remain unanswered. The heterogeneity of different vascular cells needs to be further characterized in the non-mammalian models. It also remains unclear how blood flow and hemodynamics regulate the coronary vessel formation. With the recent advancement in molecular, genetic and imaging technologies in zebrafish and other models, these critical issues can be addressed in the near future.

## Figures and Tables

**Table 1:** Origins of coronary endothelial cells in fish, avian and mammals.

	Organism	Coronary Endothelial Origin	Mechanism	Experimental Approach	Reference
**Fish**	Zebrafish (*Danio rerio*)	Angiogenic sprouting from the endocardial derived cells at the AV junction	Angiogenesis	Genetic lineage tracing, multispectral clonal analysis using *ubb:zebrabow*.	[47]
	Giant danio (*Devario malabaricus*)	Extension of a hypobranchial vessel from BA; Angiogenic sprouting from endocardium (?) at the AV junction	Angiogenesis?	Whole mount studies; BS lectin histochemistry	[48]
	Blue Gourami (*Trichogaster tricopterus*)	Extension of one or many hypobranchial vessels from BA	Angiogenesis?	Whole mount studies; BS lectin histochemistry	[48]
	Dogfish (*Scyliorhinus canicula*)	Diverticulum from the sinus venosus Epicardium derived subepicardial mesenchyme	Angiogenesis Vasculogenesis	Tissue section based morphological studies; Fibronectin imunohistochemistry	[44,45]
**Bird**	Chick (*Gallus gallus*)	Proepicardium and Epicardium derived EPDCs	Vasculogenesis	Ink/retroviral tagging /chick-quail chimera based lineage tracing studies	[28,29,30,31,32,33]
	Quail (*Coturnix coturnix japonica*)	Extension of the vessels from the sinus venosus	Angiogenesis	Tissue section based immunohistochemistry using Quail endothelium specific QH1 antibody	[49]
**Mammal**	Mouse (*Mus musculus*)	Mostly sinus venosus and endocardium; minor contribution from epicardium	Angiogenesis	Cre-LoxP based genetic lineage tracing studies	[34,35,36,37,38]
